# Progress in skin gene therapy: From the inside and out

**DOI:** 10.1016/j.ymthe.2025.03.017

**Published:** 2025-03-12

**Authors:** Mark J. Osborn, Sidharth Panda, Theresa M. Reineke, Jakub Tolar, Alexander Nyström

**Affiliations:** 1 Medical School, Department of Pediatrics, Division of Blood and Marrow Transplant and Cellular and Gene Therapy, University of Minnesota, Minneapolis, MN 55455, USA; 2 Department of Chemistry, University of Minnesota, Minneapolis, MN 55455, USA; 3 Department of Dermatology, Medical Faculty, Medical Center, University of Freiburg, 79106 Freiburg, Germany

**Keywords:** genodermatoses, dystrophic epidermolysis bullosa, gene therapy, stromal cells, gene editing

## Abstract

The skin is the largest organ of the body and forms and serves as the barrier for preventing external material from accessing and damaging internal organs. As the outward interface to the environment, it is accessible for the application of therapeutic agents and cellular and gene therapy represent attractive and promising options to treat severe genetic conditions for which palliation has long been the main stay. However, because of its barrier function, transit across and to the subdermal compartment can be challenging. This commentary examines the current approaches of cell and gene therapies for genetic skin disorders. We write this from a local and systemic “outside and inside.” perspective. Delivery from the outside encompasses topical, intradermal, and transdermal strategies for cell and vector delivery and *ex vivo* cell expansion and grafting. The inside approach details systemic delivery via infusion of cells or agents toward providing benefit to the skin. We use recessive dystrophic epidermolysis bullosa (RDEB) as a representative and paradigmatic disease to showcase these approaches as a means to highlight potential broader applicability to other conditions.

## Graphical abstract

**Figure:**
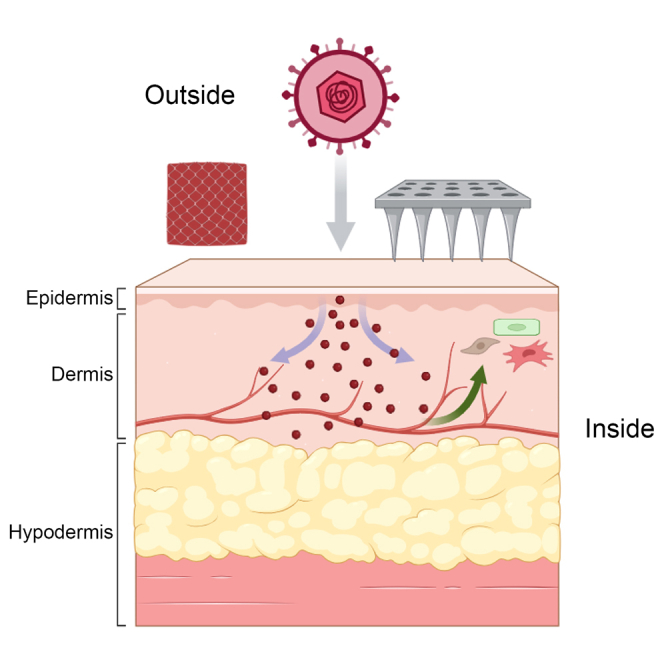

## Teaser

Gene and cell therapies hold great promise for genetic conditions of the skin to achieve more durable responses and outcomes. Osborn and colleagues highlight the state of the field using recessive dystrophic epidermolysis bullosa to discuss *in vivo* topical, transdermal, viral, non-viral, cellular, and synergistic small molecule therapeutics.

## Introduction

### Skin architecture

The skin is composed of three layers—the epidermis, dermis, and hypodermis that themselves contain architectural sub-components.^1^ The epidermis is formed by the stratum basale that is attached to the basement membrane by hemidesmosomes. Keratinocytes, melanocytes, and their stem cell precursors are the main resident cells. The stratum spinosum, in human skin, is an 8- to 10-cell-thick layer containing suprabasal keratinocytes with cytoplasmic processes that form contacts with neighboring cells through desmosomes and dendritic cells. The stratum granulosum and lucidum are ∼3–5 cell layers. The former contains granules that form bundles and adhesive glycolipids while the latter is a thicker surface found on the palms and soles of the hands and feet. The stratum corneum is the outermost layer of the epidermis and the dermis resides under it and consists of the papillary and reticular layers. Superficial is the papillary layer that contains a relatively loose extracellular matrix (ECM) with connectivity to the epidermis. The dermal layer is deeper, thicker, and less cellular and has dense collagen fiber bundles along with follicles, glands, muscles, neurons, and blood vessels. The hypodermis is subcutaneous fascia below the dermis and also contains vasculature, follicles, neurons, muscles, and glands. These layers cooperate, form, and represent the first line of barrier defense from invading agents such as chemicals, macromolecules, and pathogens and also serve to preserve hydration.

### The genodermatoses: Genetic diseases affecting the skin

The complex architecture of the skin is impacted by an equally complex array of disease states from non-genetic (wounds, burns, etc.) to genetic etiologies. A set of genetically inherited pathological conditions are the genodermatoses, a heterogeneous group of disorders that, for some, the skin manifestations are the primary and most severe complications. Treatment with cellular and gene therapies (CGTs) represents a promising therapeutic approach for genodermatoses, particularly for those in whom treating the skin and other exposed epithelium would offer significant benefits to the current standard of care, which is wound management and bandaging.

### Recessive dystrophic epidermolysis bullosa: A vanguard for the development of CGTs

Recessive dystrophic epidermolysis bullosa (RDEB) is a monogenic disease that is due to loss of or attenuated function of anchoring fibrils (AFs), a key architectural component of the skin. In skin, AFs span from the epidermal basement membrane to the papillary ECM. RDEB and impaired AF formation and function is the consequence of pathogenic *COL7A1* gene variants. *COL7A1* is a 118-exon encoding gene that occupies 32 kb on human chromosome three (3p21) that produces an ∼9 kb transcript and a 290-kDa pro-α-chain-polypeptide^2^ termed type VII collagen α1-chain. This polypeptide has a central collagenous segment and is flanked by C- and N-terminal non-collagenous domains (NC1 and NC2).^3^ Type VII collagen α1-chains assemble into a homotrimer, which constitutes one type VII collagen molecule. Extracellularly, type VII collagen molecules assume an anti-parallel dimer formation. Cleavage of the NC2 domain allows for stabilization of these anti-parallel dimers needed for the subsequent formation of functional AFs. The NC1 domains of the AF interact with basement membrane molecules (laminin-332 and type IV collagen) and the collagenous loops entrap collagen fibers. From this structure and the multi-peptide interactions, AFs serve a crucial role in skin structural attachment and integrity.^4^^,^^5^

In healthy, homeostatic human skin, type VII collagen is produced and contributed to the epidermal basement membrane zone by both basal epidermal keratinocytes and papillary-residing fibroblasts. The cellular content of the epidermis is constantly renewing through the division of basal keratinocytes followed by outward terminal differentiation—a turnover process that takes approximately 1 to 2 months.^6^ In contrast, fibroblasts in adult skin generally do not proliferate, or at least their putative renewal time/longevity is an extended one. At birth, the fibroblast density in skin is high, but as the dermis grows, which is mainly accounted for by the expansion of the ECM, the fibroblast density becomes reduced.^7^ Despite the contribution of type VII collagen by two different cellular sources, in terms of supporting skin cohesion under homeostatic conditions, either keratinocytic or fibroblastic contribution alone appears sufficient.^8^^,^^9^

Impaired function or diminished abundance of type VII collagen due to insertions, deletions, missense, substitutions, splicing, and nonsense mutations in *COL7A1*^10^ can lead to the severe RDEB phenotype characterized by external and internal disease manifestations. Cutaneous presentation is demonstrated by blistering due to mechanical stress along with milia, alopecia, pruritus, and nail dysmorphia.^11^ Chronic wounding leads to persistent wound infections, inflammation, and a heightened risk of aggressive squamous cell carcinoma (SCC).^12^^,^^13^ Additionally, RDEB presents with ocular pathology, contractures of fingers and toes, formation of pseudosyndactyly (also known as mitten deformities), esophageal strictures, and dental manifestations.^11^ Wound palliation/bandaging, frequent dental, ophthalmology, and skin screening for SCC have long been the course of care for RDEB patients.

There has been accelerated and unprecedented development in gene, genome editing, pharmaceutical, and cellular therapy approaches for RDEB over the past 2 decades that have offered treatment rather than palliation options. Allogeneic and autologous, gene-corrected cells have been employed locally and systemically. Restoration of *COL7A1* gene expression has been accomplished with small molecules, gene editing, and cDNA complementation with non-viral and viral delivery systems including Food and Drug Administration (FDA)-approved topical gene therapy vectors. Efforts continue toward enhancing delivery into the subdermal compartment using bioparticle formulations and transdermal devices. These advances are discussed here with a focus on RDEB that serves as proof of science for application more broadly.

### External therapy strategies

#### Cellular types and targets

The target cell population for therapeutic consideration and application is important, as multiple cell types are capable of producing and assembling type VII collagen. Epithelial keratinocytes and mesenchymal fibroblasts are the predominant producers of type VII collagen but other mesenchymal lineage cells can as well.^8^^,^^14^

#### Fibroblasts and keratinocytes

Fibroblasts and keratinocytes can be obtained through minimally invasive biopsies and propagated and expanded *ex vivo*. Because fibroblastic expression of type VII collagen alone appears sufficient to promote skin integrity, fibroblast-based therapies are of significant interest. Intradermal injection of allogeneic, dermally derived fibroblasts in preclinical models of RDEB supported their role as an effective vehicle for type VII collagen delivery to the epidermal basement membrane zone.^15^^,^^16^ Subsequent trials with intradermal injections of allogeneic fibroblasts showed variable efficacy, but in general, the level of type VII collagen restoration was low.^17^^,^^18^^,^^19^^,^^20^ This could partially be due to host reactivity against the allogeneic fibroblasts.

Keratinocytes are the predominant skin cell of the epidermis and can be classified by subtype. Three broadly conventional keratinocyte populations exist: basal, spinous, and granular based upon which layer of the epidermis they are found. Similar to fibroblasts, keratinocytes participate in wound healing and fibrosis^21^ and can also be derived and expanded, making them amenable for *ex vivo* gene correction strategies that have progressed to the clinic using autologous, gene-corrected grafts.

### Restoring *COL7A1* gene expression: Adding back and locus targeting

#### Integrating vector gene therapy

To avoid immune-related clearance of allogeneic cells, correction of patient-derived fibroblasts and keratinocytes using lenti- or retroviruses has been pursued for adding back a functional *COL7A1* cDNA element.^15^^,^^22^^,^^23^ Georgiadis et al. described a GMP-compatible self-inactivating (SIN) lentivirus where a codon-optimized *COL7A1* cDNA was expressed under the control of the human phosphoglycerate kinase (PGK) promoter.^22^ Transduction of autologous RDEB fibroblasts led to supranormal type VII collagen production and supported AF formation at the epidermal basement membrane zone of RDEB skin grafts on immune-deficient mice.^22^ In a clinical trial, intradermal injection of autologous fibroblasts transduced with a *COL7A1* SIN lentivirus overall led to higher deposition of type VII collagen at the epidermal basement membrane zone compared with what was reported in trials using allogeneic wild-type fibroblasts.^17^^,^^19^^,^^24^ Since these studies were not conducted head-to-head and involved a small number of patients, definitive conclusions are challenging to draw. However, the relative ease of obtainment and culture of autologous cells for use with gene/protein restoration continues as a promising therapeutic avenue.

**Figure 1:**
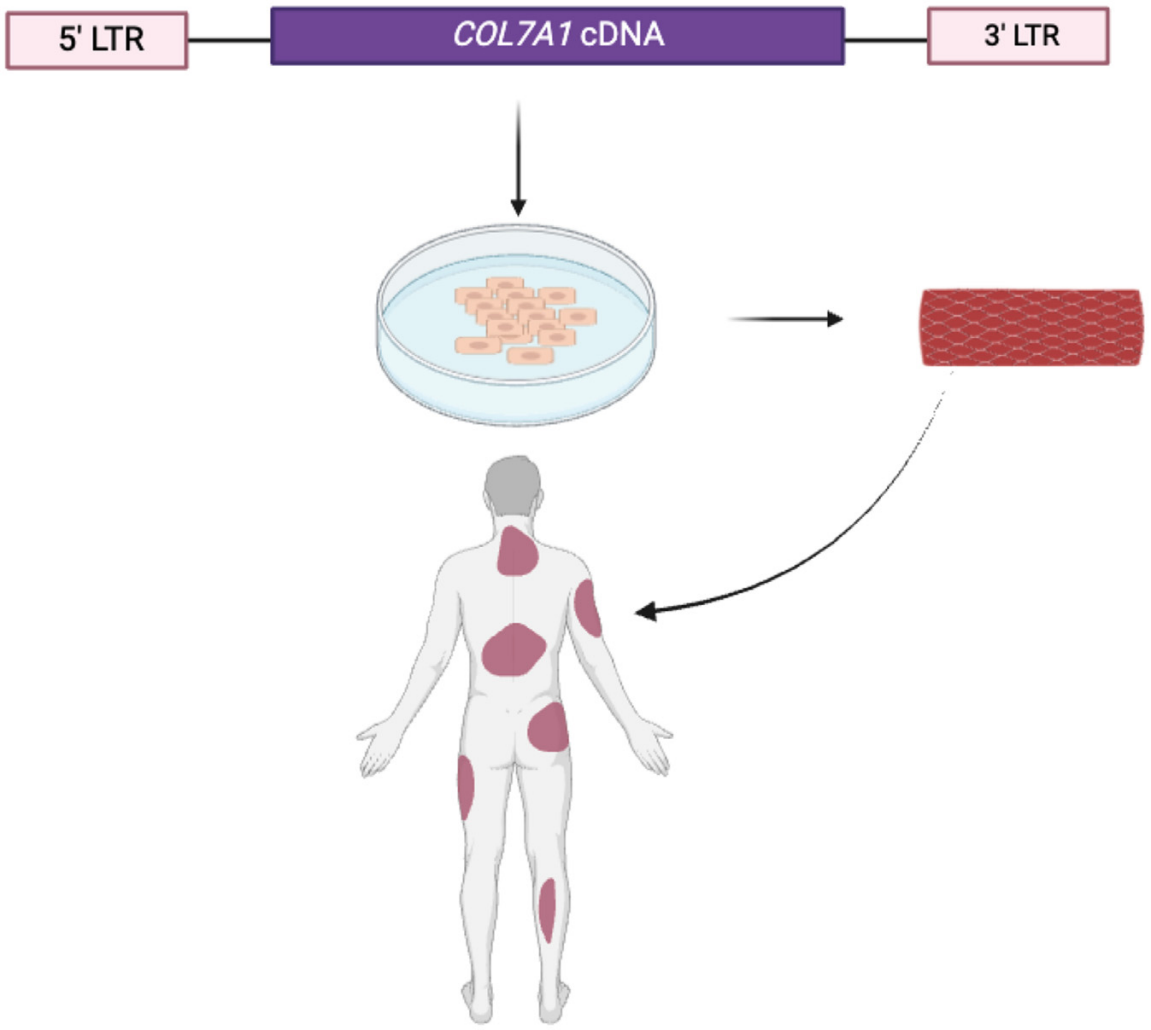
*Ex vivo* gene therapy for RDEB A retroviral vector was employed to transduce autologous RDEB keratinocytes for *ex vivo* expansion into skin sheets that were then grafted onto wounded areas of patients.

In a phase I trial (NCT01263379), autologous keratinocytes were harvested, complemented with a *COL7A1* retroviral vector, expanded into grafts, and surgically grafted onto patients (Figure 1). The vector was composed of a gibbon ape leukemia virus pseudotyped envelope and a full-length version of the *COL7A1* cDNA with a moloney leukemia virus long terminal repeat (LTR) facilitating expression.^25^ Vector copy number (VCN) analysis showed 0.8 copies/genome and 70% transduction efficiency and the cells were then expanded into approximately 35 cm^2^ sheets that were grafted onto wounded areas of patients.^25^ Presence of type VII collagen in grafted wounds was demonstrated in nine of 10 patients at 3 months and this frequency decreased to five of 12 patients at 1 year. Gene-corrected cellular grafts resulted in wound healing in 21 of 24 graft sites with the remaining three showing extensive blistering and erosion.^25^

Evaluation of long-term effects of the single-center phase 1/2a portion of this study followed patients (*n* = 7) for 2 to >5 years.^26^ At 1 year, 26 of 38 wounds that received grafts had >50% healing and at year 2 the wound healing frequency was 27 of 38.^26^ Longer-term analyses (4–8 years) showed that 21 of 30 treated sites at year 5 had >50% would healing compared with baseline.^27^ Importantly, no serious adverse events were reported (mean of 5.9 years) as evidenced by a lack of replication competent retrovirus infections, no long-term autoimmunity, and no incidence of SCC.^25^^,^^26^^,^^27^ The results from this clinical trial showed improved healing and a lack of adverse events, indicating a favorable safety profile; however, the therapy represents a labor- and infrastructure-intensive treatment algorithm. The derivation, transduction, and expansion of autologous patient cells require specialized vector production and cell manufacturing facilities. Further, the transport of grafts to an advanced medical center with the need for general anesthesia, surgical application, and postoperative recovery requires dedicated medical experts and for the patient(s) and family to travel and temporarily relocate, making it difficult to offer to a broad patient population located around the globe.

#### Topical and transdermal therapy

Toward economizing access both with regard to delivery of the therapeutic as well as offering feasible treatment options for a wider community, topical and transdermal gene therapy strategies for dystrophic epidermolysis have been and continue to be developed. Topical delivery is challenged by the natural function of the stratum corneum of the skin as a barrier, limiting efficient transdermal uptake of molecules larger than 500–600 Da.^28^ To circumvent such challenges, delivery to wounds or areas with epidermal breaches can be considered, as well as inducing epidermal microbreaches through, e.g., laser pulsation, poration, and/or transit with nanoparticles.

To bypass the stratum corneum barrier for more efficient transdermal delivery, transfollicular delivery through hair follicles and glands has been pursued.^29^ Utilizing entry through hair follicles may also allow for deeper dermal distribution of drugs and for penetration of molecules larger than 500–600 Da in size. However, these efforts are limited by the preference for the uptake of lipophilic molecules through this route and also by the number of hair follicles, which greatly vary across anatomical sites and individuals. For example, this would be a limitedly effective delivery approach on the palms, soles, trunk, and neck. Efficient topical administration of proteins or vectors to skin in a uniform fashion over broad surface areas remains a need and systemic and intradermal administration remains the mainstay for unbreached skin.

### Viral vectors

#### Lentivirus

Intradermal injection of *COL7A1* cDNA lentivirus has demonstrated the restoration of type VII collagen abundance at the epidermal basement membrane in preclinical RDEB models including RDEB skin equivalents in immunocompromised and type VII collagen knockout mice.^30^

#### Herpes simplex virus type 1 vector

Herpes simplex virus type-1 (HSV-1) has also been pursued as a delivery vehicle for the *COL7A1* gene. As a pathogen, HSV-1 requires a breach (e.g., macro- and microabrasions) in the epidermal barrier to infect the skin and mucosa where it can enter, replicate, translocate to neuronal cells, establish latency as a non-integrating, episomal species, and later reactivate.^31^ The HSV-1 virion contains a large, double-stranded DNA genome core of ∼84 genes over 152 kb that is encapsulated by a tegument layer and an outer envelope icosahedral capsid.^32^ The constituent genes are categorized as accessory or essential with envelope glycoproteins, replication factors, capsid, and DNA cleavage being essential gene components. Accessory genes encode metabolic, regulatory, structural, and protective peptides, some of which are dispensable and have been modified for *in vitro* and engineering use but have not been observed in patients.^33^ Both accessory and essential genes cooperate to generate infectious particles with replication competency; however, non-replicating particles with infectivity have been developed through engineering that enable the use of HSV-1 as a gene therapy/delivery vehicle.^34^^,^^35^^,^^36^ ICP4, encoded by the RS1 gene, is a regulator of early and late viral genes and has been deleted to result in non-replicating HSV particles that can infect cells and mediate sustained transgene expression.^35^^,^^37^

**Figure 2:**
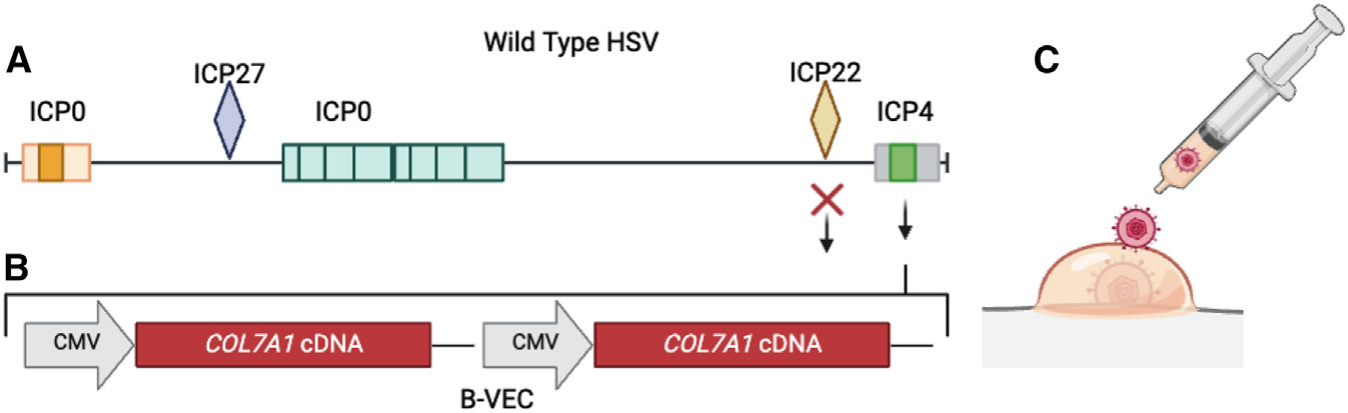
HSV-1 vector design and delivery (A) The wild-type HSV genome is diagrammed with Infected Cell Protein (ICP) genes shown that are involved in viral replication. (B) The engineering strategy by Gurevich employed two copies of the *COL7A1* cDNA driven by the cytomegalovirus (CMV) promoter that were inserted into the ICP4 region of HSV-1. An additional deletion of ICP22 (red “x”) was also created. (C) Delivery. HSV-1 (B-VEC) can be applied via droplets to the skin using a syringe.

HSV-1 is a uniquely suited vector for conditions of the skin such as RDEB, as it accommodates large genetic cargo and has intrinsic skin infectivity properties. Gurevich and colleagues^38^ leveraged these aspects to package two copies of the *COL7A1* gene within non-replicating HSV-1 particles and applied them topically to wounds to restore *COL7A1* expression (Figure 2). This was accomplished by inserting the *COL7A1* expression cassette into the ICP4 gene and additionally deleting the ICP22 gene. This vector, beremagene geperpavec (B-VEC), underwent proof of principle/validation studies and restored type VII collagen synthesis in RDEB patient keratinocytes and fibroblasts prompting further evaluation in a murine model of RDEB. Three doses (4.6 × 10^7^ plaque-forming units [p.f.u.] per 50 μL per injection) was administered to *Col7a1*-deficient mice (*n* = 27 and *n* = 9 vehicle controls) resulting in contiguous deposition of type VII collagen at the epidermal basement membrane and assembly into AFs. These data supported human patient clinical translation where, in phase 1 and 2 trials, B-VEC was administered topically to RDEB patient wounds. The clinical trial (NCT03536143) structural profile was arrayed to include a safety arm of *n* = 9 RDEB patients and an efficacy phase with *n* = 18 B-VEC treated and *n* = 10 placebo treatment groups. Doses were based on size of wounds/surface area and ranged from 1 × 10^8^ p.f.u. of B-VEC per dose to 8 × 10^8^ p.f.u./dose for two treatment cycles of ∼20 treatments over ∼25 days. The primary and secondary study criteria for this cohort were met, with AF deposition, wound surface area reduction, duration of closure, and time to wound closure showing improvement compared with placebo.^38^ Results from a follow-on phase 3 trial were reported by Guide et al.^39^ (NCT04491604) that was double blinded with intrapatient randomization and wound treatment with B-VEC or placebo. The primary endpoint was complete wound healing at 6 months and patients were dosed with B-VEC from 4 × 10^8^ to 1.2 × 10^9^ p.f.u. At 3 months, healing was observed in 71% of wounds (20% in placebo) and at 6 months, 67% of B-VEC treated wounds showed complete healing compared with 22% with placebo.

Because RDEB patients manifest with pathology in multiple organ systems (skin, eye, viscera) and B-VEC can be administered topically onto wounds, a patient with cicatrizing conjunctivitis was treated with 5 × 10^9^ p.f.u. in a study led by Tovar Vetencourt.^40^ To accomplish this, a bandage contact lens was placed followed by B-VEC three times per week for 2 weeks and then once weekly for a total of 19 doses. At 3 months, complete healing of the corneal epithelium and improved visual acuity was described as measured by optical coherence tomography and slit-lamp examination.

B-VEC is now FDA approved (Vyjuvek) and was recently recommended for approval by the European Medicines Agency. It is an exciting addition to the treatment options for patients with RDEB and HSV-1 is an impactful tool in the gene therapy toolbox for broader skin condition treatment. Noteworthy from these RDEB clinical trials was a paucity of serious adverse events. Side effects included fever, rash, itching, and presence of antibodies at baseline. Importantly, significant immunological side effects were not reported,^39^ which may be in part due to the vector design and innate immune-evading properties of HSV-1. The ICP47 gene inhibits antigen processing^41^ and engineered HSV genome deletions (ICP4 and ICP22) that minimize vector-related inflammation promote prolonged expression and avoidance of vector or transgene immune-related side effects.^34^^,^^37^^,^^42^^,^^43^^,^^44^

### *COL7A1* gene expression and gene editing

#### Locus elemental control

The exogenous gene regulatory elements and *COL7A1* expression levels and duration for gene therapy is an important consideration. Integrating vectors result in transmission of the vector to progeny cells and a sustained and often supraphysiological expression profile; however, risks of insertional mutagenesis are significant particularly in the RDEB pre-malignant phenotype.^45^^,^^46^^,^^47^ Non-integrating HSV-1 vectors mitigate the genotoxic risks of integrating vectors; however, the transient nature of the platform can limit gene-expression duration. In clinical trials to date, repeat doses of B-VEC were often employed and the predicted cost to patients is >$10,000,000 over their lifetime.^48^

Many strategies for rescuing *COL7A1* gene expression with gene therapy use non-native elements that produce supranormal levels of the type VII collagen.^22^^,^^49^^,^^50^ Because *COL7A1* expression is governed by internal regulatory type VII collagen mechanisms^51^^,^^52^ and influenced by external stimuli,^53^ preservation of endogenous elements and expression patterns and levels is desirable.^52^^,^^54^ Toward this, the retroviral vector design by Titeux^50^ incorporated elements from the *COL7A1* promoter in a SIN design with the region −524 to +92 in orientation to the initiating methionine. However, the integrating properties of viral vectors remains a compounding risk. A strategy that can correct disease driver mutations and retain control of endogenous elements is afforded by locus-specific genome editing.

#### Genome editing with programmable DNA binding platforms

**Figure 3:**
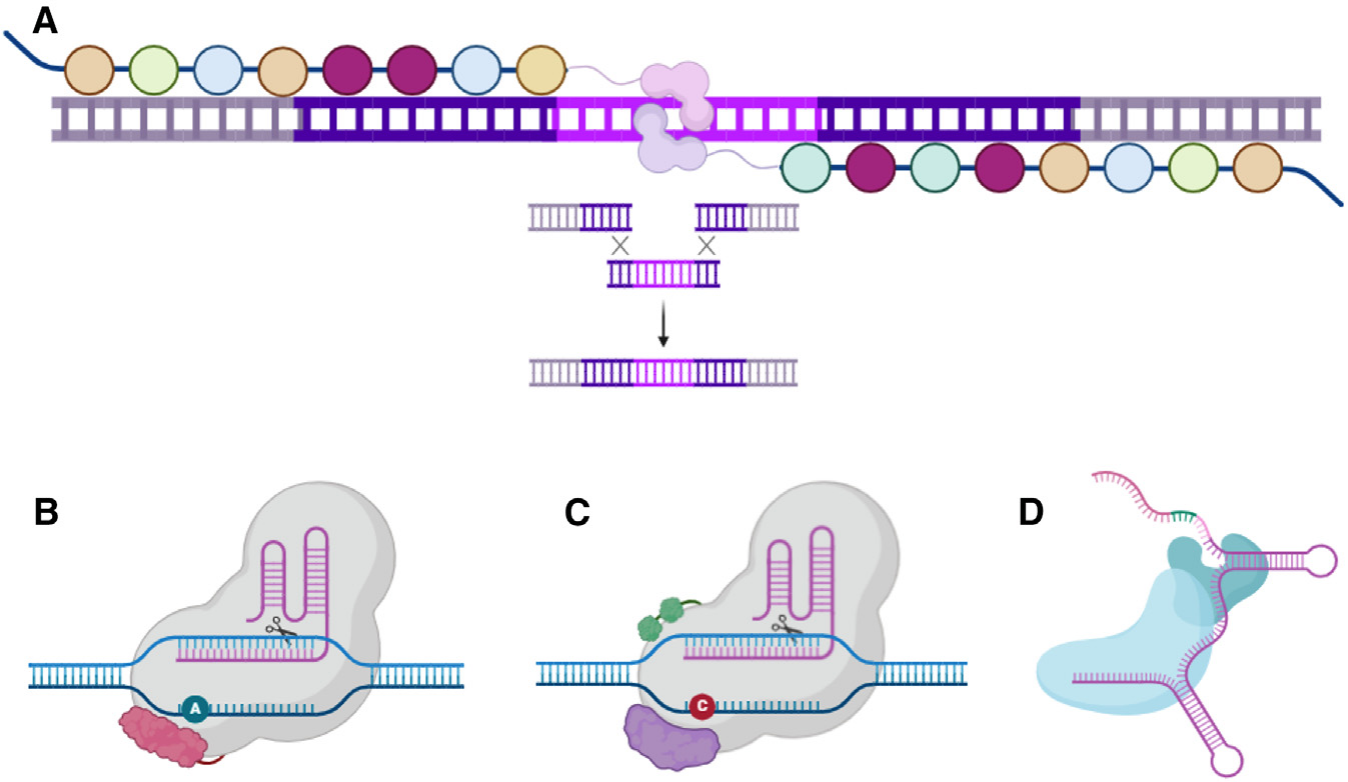
Gene editing for skin cells (A) TALEN architecture and HDR. TALENs are composed of arrayed DNA binding elements (colored bubbles) with repeat variable diresidues that are specific for a single base of DNA. Tethered to the DNA binding repeats is a *Fok*I nuclease that generates a double-stranded DNA break.^59^ In the presence of an exogenous repair template, the break can undergo homology-directed repair and insertion of user-defined sequences. (B–D) Genome editing platforms. Adenine (B), cytosine (C), and prime editor (D) architectures are shown. Each employ a Cas9 nickase for DNA binding. ABE employs a natural or evolved adenine deamination domain to convert A>G (B).^76^^,^^77^^,^^80^ CBE contains a cytosine deaminase along with uracil glycosylase inhibitors (UGI) to promote C>T editing (C).^77^ The prime editor employs a reverse transcriptase enzyme from nature or laboratory evolved versions that use an engineered prime editor guide RNA (pegRNA) as a template for RNA-dependent DNA synthesis (D).^78^^,^^90^^,^^91^ The pegRNA can accommodate all 12 possible base combinations/substitutions.^185^

Homology-directed repair (HDR) has transformed the biological sciences and employs an extrachromosomal, homologous donor molecule with user-defined sequences that can be incorporated into the genome at low frequencies.^55^ HDR efficiencies can be increased in the presence of a double-stranded DNA break (DSB) created by DNA binding proteins.^56^ Spurred by this, designer nucleases based on zinc finger (ZF) DNA binding proteins were developed that could introduce site-specific DSBs and induce repair from a donor *in trans* to modify targeted sequences in the genome.^57^^,^^58^ These foundational studies further prompted design and platform advances that economized and made genome editing more broadly available. Transcription activator-like effector nucleases (TALEN) possess repeat binding domains that have a 1:1 relationship with a nucleotide such that rationale engineering for binding a user-defined sequence could be accomplished (Figure 3A).^59^ With TALENs and HDR, we corrected an inactivating mutation in the *COL7A1* gene in RDEB patient-derived fibroblasts that were differentiated into induced pluripotent stem cells (iPSCs) and restored type VII collagen *in vitro* and *in vivo*.^60^ TALEN construction, while more amenable for broader scientific community use compared with ZFNs, still requires cloning of repetitive elements and the generation of candidates can be challenging.^61^

The clustered regularly interspaced short palindromic repeats (CRISPR)-Cas9 system offers facile design principles and the Cas9 nuclease has been employed by multiple groups for *COL7A1* targeting and correction using single- or double-stranded repair donor templates.^62^^,^^63^^,^^64^^,^^65^^,^^66^^,^^67^ While DSBs stimulate HDR, our correction frequencies for both TALEN and CRISPR-Cas9 nucleases was sufficiently low that it prompted donor design parameters for the inclusion of a selectable marker that had to be removed by addition of Cre recombinase.^60^^,^^68^ The employment of drug selectable markers and need for a second step to remove them is burdensome and difficult to advance for efficient engineering in support of translation. Furthermore, DSBs are repaired by competing HDR and non-homologous end-joining (NHEJ) pathways that can result in mixed allelic purities of HDR with the accompanying presence of insertions and deletions (indel) from NHEJ.^69^^,^^70^ Like viral vectors, nuclease-based gene targeting carries risk, as DSBs are toxic and trigger p53 activation^71^ and destabilizing genome events such as chromothripsis.^72^ Additionally, the complexity/heterogeneity of the target site compared with overlapping/partially homologous sequences that may be present elsewhere in the genome can lead to gRNA-dependent Cas9 off-target events.^73^ Our studies with TALENs using linear amplification-mediated PCR did not identify off-target events.^60^ More recent efforts using CAST (chromosomal aberrations analysis by single targeted linker-mediated PCR sequencing)-seq^74^ in EB-patient-derived keratinocytes that were edited with Cas9 nuclease identified chromosomal rearrangements^75^ and highlight the importance of sensitive off-target analytical methods and preserving the integrity of the genome, particularly in RDEB where malignant transformation is highly prevalent.

#### DNA base and prime editing

Toward maximizing the potential and employing the concepts of locus-specific targeting while minimizing DSBs and removing the need for donor molecules and, potentially selection, emerging platforms such as the DNA base editor (BE)^76^^,^^77^ (Figures 3B and 3C) and prime editor (PE)^78^ (Figure 3D) represent promising tools.

BE and PE each employ versions of Cas9 that retain programmable DNA binding but rather than mediating DSBs, they introduce single-stranded nicks due to inactivation of one of the Cas9 HNH or RuvC endonuclease domains. They additionally contain functional domains of either a deaminating enzyme (BE) or a reverse transcriptase (PE). BEs contain either an adenine deaminase (ABE) that can facilitate conversion of A⋅T to G⋅C or a cytidine deaminase (CBE) capable of C⋅G to T⋅A DNA transition modifications. PE, ABE, and CBE have been applied for *COL7A1* gene correction in RDEB patient cells. Using an early version of ABE (ABE 7.10) we showed that electroporation of mRNA into fibroblasts could accomplish correction of mutations in primary fibroblasts and iPSCs from two RDEB patients.^79^ Sheriff and colleagues, using an evolved ABE (ABE8e^80^^,^^81^), achieved correction rates of >90% without need for a donor molecule or selection.^82^ More recently developed BE platforms can also mediate transversions^83^^,^^84^^,^^85^^,^^86^^,^^87^^,^^88^; however, the mutational constellation of *COL7A1* is such that transitions, deletions, transversions, and insertions are reported.^10^

With an engineered reverse transcriptase that primes and synthesizes off an engineered prime editor guide RNA (epegRNA) template with an encoded user-specified sequence, PE enables the installation of all base substitutions, insertions, deletions, and combinations thereof.^78^ With PE, Hong et al. corrected two *COL7A1* patient mutations and derived skin equivalents that showed deposition of type VII collagen and AF formation in mice *in vivo*.^89^ Newer iterations of the PE offer greater activity and smaller architectures,^90^^,^^91^ and both BE and PE enable sequence modification without the need for a donor template and mediate editing events with lower rates of DSB and therefore NHEJ indels than occurs with nucleases. For rare disease indications and/or genes with a large and diverse repertoire of disease-causative mutations such as is observed in RDEB/*COL7A1*, the development of patient-specific reagents for individualized therapy represents a potential hurdle due to the variability in activity of individual reagents and the potential high cost and lengthy regulatory process to obtain personalized clinical grade reagents.

A potential solution is to merge the concepts of gene therapy and editing. Gene therapy offers the ability to create a cDNA vector system applicable across most or all patients; however, these vectors can either be transient requiring re-dosing or integrate at semi-random and pose genomic insertional mutagenic concerns. Further, the inclusion of non-native gene regulatory elements and sustained transgene expression at supranormal levels may not be indicated in every instance. Gene editing enables the retention of locus-specific control at the expense of broad applicability. Toward resolving this gap, CRISPR platforms that enable precision installation of large genetic cargo, such as the ∼9 kb *COL7A1* cDNA, represent exciting technologies. Advances toward this include programmable addition via site-specific targeting elements (PASTE),^92^ prime-editing-assisted site-specific integrase gene editing (PASSIGE and evolved, engineered [ee]PASSIGE),^93^^,^^94^ and CRISPR-associated transposases^95^ that all facilitate large genetic element insertion without the need for/introduction of DSBs. Under this concept, a cDNA could be installed, without DSBs, proximal to the promoter region and upstream of reported mutations to achieve the broad applicability of gene therapy with preservation of the in-place expression regulators afforded by locus-specific targeting.

### Delivery: Viral, non-viral, physical, systemic, and immune responses

Vital organs are protected in layers of connective tissue, muscle, bone, and the skin. The skin serves as the interface with the external world and is the first line of defense as a protective barrier from pathogens, environmental, and physical threats. Skin tight junctions function for water retention and exclusion of macromolecules and represent a challenge for the delivery of therapeutic agents. Above we discuss the agents and platforms that can restore *COL7A1* expression and below we discuss cellular, viral, non-viral, and physical options that can facilitate delivery of therapeutic payloads to and through the skin.

#### Natural affinity/tropism

Cells and viral vectors possess migratory and tropism properties that can be leveraged to facilitate delivery of therapeutic genes/peptides to predictable compartments. The viral envelope glycoprotein D (gD) on HSV-1 interacts with skin cellular receptors nectin-1 and herpesvirus entry mediator (HVEM).^96^ Keratinocytes, the major cell type of the epidermis, and fibroblasts, the major resident cells of the dermis, show expression of each receptor.^96^ Comparatively, keratinocytes show higher expression of nectin-1 and fibroblasts HVEM and studies support nectin-1 as the key facilitator of HSV-1 entry.^96^^,^^97^ Wild-type HSV transits to the nucleus where it replicates and new virions infect proximal neuronal cells with retrograde trafficking to the nucleus where latency is established by silencing of viral genes, save for the latency-associated transcript (LAT) gene that remains active.^98^ Reactivation can occur where infectious particles re-infect epithelial cells causing cell death/lesions.^31^ B-VEC retains some and lacks some of the properties of wild-type HSV. B-VEC HSV particles infect epithelial cells but do not express LATs and therefore cannot establish latency and limits concerns of replication and reactivation.^99^ B-VEC HSV does, like wild type, express the ICP47 gene that inhibits antigen presentation and immune recognition.^38^^,^^39^ This allows for diminished transgene responses and repetitive administration and while HSV-1 and anti-collagen type VII antibodies have been detected in patients, these immune responses, in results to date, have not altered the therapeutic trajectory and potential.^100^ This is a critical aspect and benefit to HSV-1 in comparison with and in contrast to other viral systems (e.g., retroviral and adenoviral) that can trigger immune responses that can be severe.^101^^,^^102^^,^^103^ However, since patients develop type VII collagen antibodies, careful long-term monitoring of this relatively new treatment modality will be needed to exclude a reduction in efficacy or even the occurrence of pathological autoimmunity toward type VII collagen.

#### *In vivo* delivery

HSV-1 can be applied topically *in vivo* and while highly effective at transducing cells, still requires a breach in the stratum corneum in order to access cells to infect. As such, HSV-1 can deliver *COL7A1* to active and open wound areas for wound healing but may be less well suited for preventing wounds in areas of the skin that are intact at the time of treatment but subject to wounding.

Non-viral platforms such as with cDNA or gene editing platforms require additional consideration for *in vivo* delivery as they possess no innate cellular entry properties like viruses do. This represents a challenge for *in vivo* gene editing approaches, and we discuss below the use of encapsulating/carrier molecules and devices for nucleic acid (cDNA, base, and prime editors) to facilitate transdermal transfer *in vivo*.

#### Nanoparticles

Viruses encapsulate nucleic acids in capsids and envelopes that shield them from degradation and also enables cellular entry. Lipid and biopolymeric nanoparticles are promising surrogates for viral nucleic acid packaging and delivery. Highly branched poly(β-amino esters) (HPAEs) are biodegradable polymers with reversible charge properties that promote nucleic acid binding and enable modularity and customization for tailored tissue/gene delivery including the skin.^104^^,^^105^^,^^106^ Zeng and colleagues applied HPAEs to encapsulate a minicircle DNA with a *COL7A1* transgene sequence and performed intradermal injections or topical application in mice and showed type VII collagen at the basement membrane zone.^107^^,^^108^

**Figure 4:**
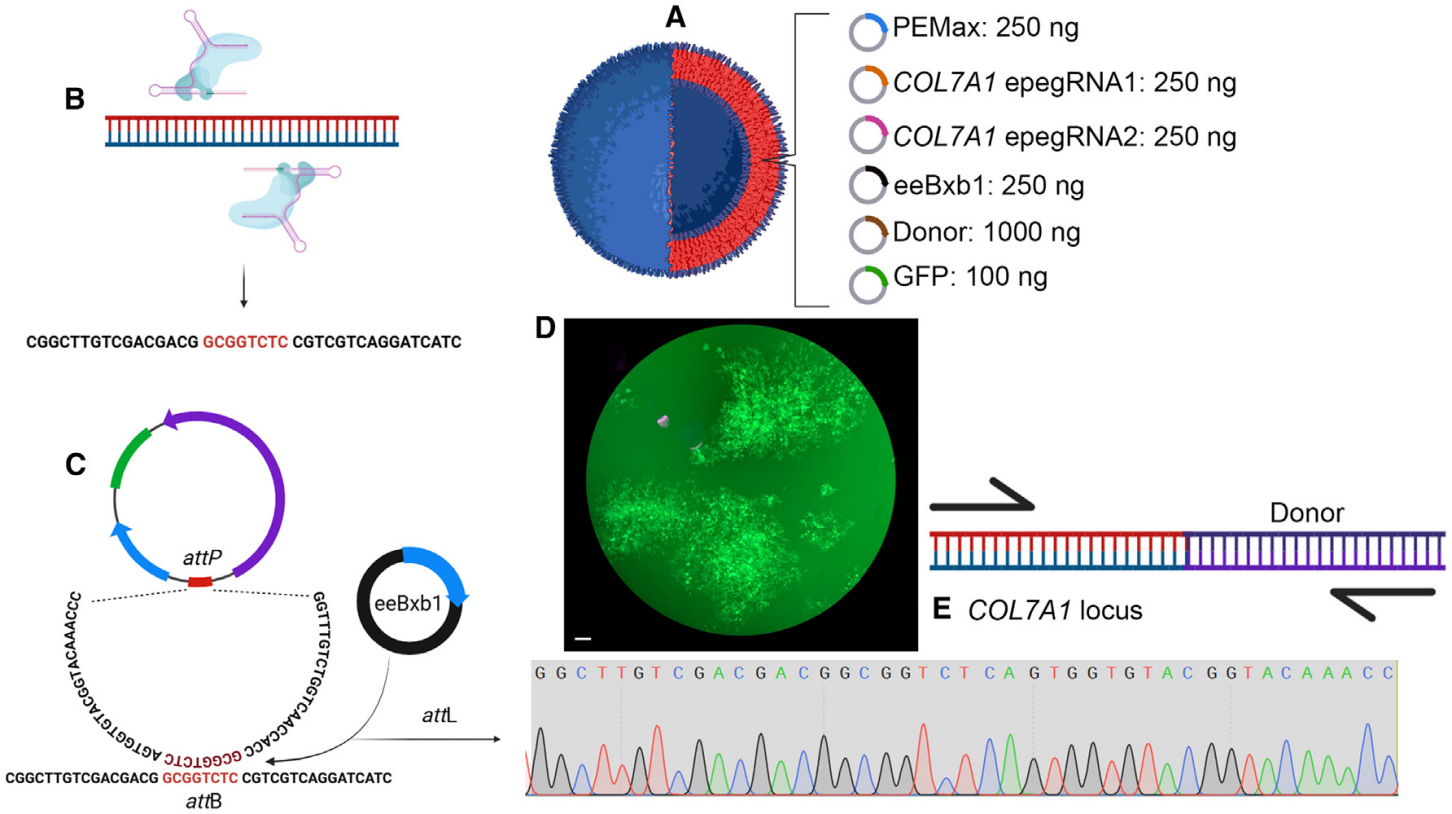
Nanoparticle delivery (A) The plasmids required for prime-editing-assisted site-specific integrase gene editing were encapsulated in micellar nanoparticles with a cationic modifier (chemical structure at left; the image for which was created in ChemDraw, Revvity Signals, Waltham, MA) and the amount of plasmid, in nanograms, is detailed at right. (B and C) Mechanism of eePASSIGE. Prime editor guide RNAs with attachment (att) sites are targeted to opposite strands of a target locus (e.g., *COL7A1*) and install the att site into the genome (B). In the presence of the Bxb1 recombinase and a donor molecule with an att site, a unidirectional genomic recombination event is mediated converting the *att*B and *att*P to an *att*L (C). (D) Nanoparticles with eePASSIGE and GFP plasmids were delivered to 50,000 HEK293s in a 24-well dish using serum-free media for 4 h and then regular growth media was added with culture for 96 h. A photomicrograph using an inverted fluorescent microscope (4× objective) is shown and the white bar is 100 μm. (E). Screening. Treated cells were harvested at 96 h and genomic DNA was screened by PCR using a *COL7A1* locus-specific primer and a primer within the donor as an “inside-out” strategy. The PCR product was sequenced and showed the post-recombination *att*L site, shown shaded in gray in the Sanger sequence chromatogram.

Lipid nanoparticles (LNPs) range in size, charge (cationic lipids vs. neutral lipids vs. anionic lipids, phospholipids), and cargo capacity, and can be attached to specified targeting ligands.^109^^,^^110^^,^^111^^,^^112^^,^^113^^,^^114^^,^^115^^,^^116^^,^^117^^,^^118^^,^^119^ LNPs are potent, well tolerated with reduced immunogenicity, capable of base and prime editor delivery, scalable, and safe.^109^^,^^110^^,^^111^^,^^112^^,^^113^^,^^114^^,^^115^^,^^116^^,^^117^^,^^118^^,^^119^^,^^120^^,^^121^^,^^122^ Guri-Lamce^123^ et al. showed that LNPs could deliver ABE to RDEB fibroblasts *in vitro*, achieving >80% *COL7A1* gene editing efficiencies that establish important proof of concept for expanded delivery options for topical application of gene editors. Toward expanding this concept for delivering multi-cargo payloads, we have undertaken efforts to employ machine learning to inform the generation cationic micelle nanoparticles (MNPs) from diblock copolymers for cellular delivery. We employed one of these MNP candidates, A7 (S.P. and T.M.R., unpublished data), to encapsulate and deliver six plasmids for evolved, engineered PASSIGE: the DNA prime editor, an evolved and engineered Bxb1 recombinase, two epegRNAs, a donor molecule, and a GFP reporter (Figure 4A). The epegRNAs install an attachment B (*att*B) site into the *COL7A1* locus (Figure 4B) and the large serine Bxb1 recombinase mediates a unidirectional recombination between the genomic *att*B and an attachment P (*att*P) on a donor molecule (Figure 4C). With the A7 MNP, we delivered the plasmids to HEK293T cells and observed GFP expression (Figure 4D). PCR using a *COL7A1* locus and donor-specific primer with sequencing showed the post-recombination *att*L footprint (Figure 4E). The plasmid donor we employed was 9.4 kb, which approximates the size of the 8.9 kb *COL7A1* cDNA and provides proof-of-concept data for the use of MNP as a multicomponent, large-element delivery vehicle with the ability to install cargo at the 5′ end of the *COL7A1* locus.

The use of chemical encapsulation such as with LNP, MNP, and HPAEs offer further potential because they have less large cargo capacity and lack of viral host or envelope proteins that may be immunogenic.

#### Devices for delivery

Encapsulating cargo in particles and delivering them transdermally using mechanical disruption of the epidermal barrier may hold synergistic potential for therapeutic molecules. In a study led by You et al.,^124^ the type I collagen (*COL1A1*) mRNA was encapsulated in extracellular vesicles and delivered to the skin of mice in a photoaging model using a microneedle array. Similar to HSV-1, this strategy would represent a transient one because HSV-1/mRNAs are extrachromosomal. Further efforts have employed LNPs^125^ or polymeric nanoparticles^126^ for CRISPR-Cas9 mRNA or ribonucleoprotein complexes representing a potential strategy to correct genes *in situ*, *in vivo* that retains endogenous gene regulation in a more durable fashion, which would potentially diminish the need for repetitive delivery. As has been pursued *ex vivo*, editing strategies to date for *in vivo* and transdermal delivery represent a more individualized approach but establish proof of principle and warrant further development of more broadly applicable editing platforms for topical, *in vivo* use.

#### Inside

##### Infusion for systemic delivery

*In vivo* injection and infusion of peptides, proteins, and/or cells represents a strategy for treating conditions of the skin.

#### Recombinant protein

Intradermal injection of purified type VII collagen protein was shown in preclinical models to restore AFs in RDEB skin and in neonatal animals showed systemic biodistribution.^127^^,^^128^ Because recombinant type VII collagen by itself does not actively promote thrombosis, as other fibrillar collagens might, it can safely be administered through intravenous injections. Such injections with systemic distribution have shown variable efficacy in restoring skin and epithelial cohesion in collagen VII-deficient mice.^129^^,^^130^^,^^131^ However, the efficacy was reduced in RDEB mice with more advanced dermal changes,^129^ illustrating that disease-related dermal changes must be considered as modulators of treatment efficacy. Such changes may include altered distribution and modifications of interaction sites for the restored protein, as exemplified by variable efficacy of type VII collagen introduction in restoring skin cohesion in these cases.

#### Hematopoietic and mesenchymal lineage cell therapy

Hematopoietic progenitors and mesenchymal stromal cells (MSC) have been pursued for EB cellular therapy for infusion locally and intravenously for biodistribution via the vasculature. Transplant with the true hematopoietic stem and progenitor cell fraction showed engraftment and enhanced survival in mice^132^ that was used for subsequent clinical pursuit. In humans, allogeneic bone marrow transplantation improved skin appearance and reduced wound burden in recipients with RDEB; however, significant complications were possible due to immune-related side effects from allogeneic cells.^133^ As an alternative, MSCs—a mesenchymal lineage cell type with high regenerative potential and immunomodulatory properties—extracted from various tissues, including bone marrow and skin, have been tested. Early studies in humans demonstrated that intradermal injection of 0.5 million allogeneic MSCs promoted the healing of ulcerated skin in RDEB and facilitated type VII collagen deposition at the epidermal basement membrane zone.^134^ Studies in RDEB mice further supported that intradermally injected MSCs at high concentrations can directly contribute to type VII collagen deposition at the epidermal basement membrane zone.^14^ Importantly, these studies also revealed that MSC application promoted wound healing, with some effects appearing to be independent of type VII collagen. Instead, these effects were attributed, in part, to the ability of MSCs to modulate cellular and inflammatory activities through paracrine signaling.^14^ In studies using RDEB mice, a transient presence of MSCs in injected skin and wounds was observed; however, subsequent studies revealed extended residence and proliferation of intradermally administered wild-type or engineered RDEB MSCs in RDEB skin grafts.^135^^,^^136^ This highlights the potential to promote permanent repopulation of injected MSCs in RDEB skin under optimized conditions, providing a renewable cellular source of type VII collagen.

Building on this, studies using MSCs extracted from RDEB blister fluids and genetically modified to express high levels of *COL7A1* and type VII collagen under the control of the CAG promoter revealed the influence of administration configurations on the potential for long-term engraftment.^137^ In these studies, intradermal injection into skin grafts from type VII collagen-deficient mice resulted in only transient engraftment of the administered MSCs in the skin. In contrast, when administered into blisters or as cellular sheets applied to wounds, long-term engraftment of MSCs was achieved.^137^

An MSC population with enhanced modulating activity are dermal MSCs marked by ATP-binding cassette sub-family B member 5 (ABCB5) positivity. When injected into type VII collagen-deficient mice, these cells exhibited significant disease-modulating effects. This finding supported clinical testing in RDEB patient cohorts.^138^ In the first trial (NCT03529877; EudraCT 2018-001009-98), repeated infusions of ABCB5^+^ MSCs met safety and tolerability endpoints and demonstrated disease-modulating effects, including reductions in EB-specific clinical scores and itch.^138^

**Figure 5:**
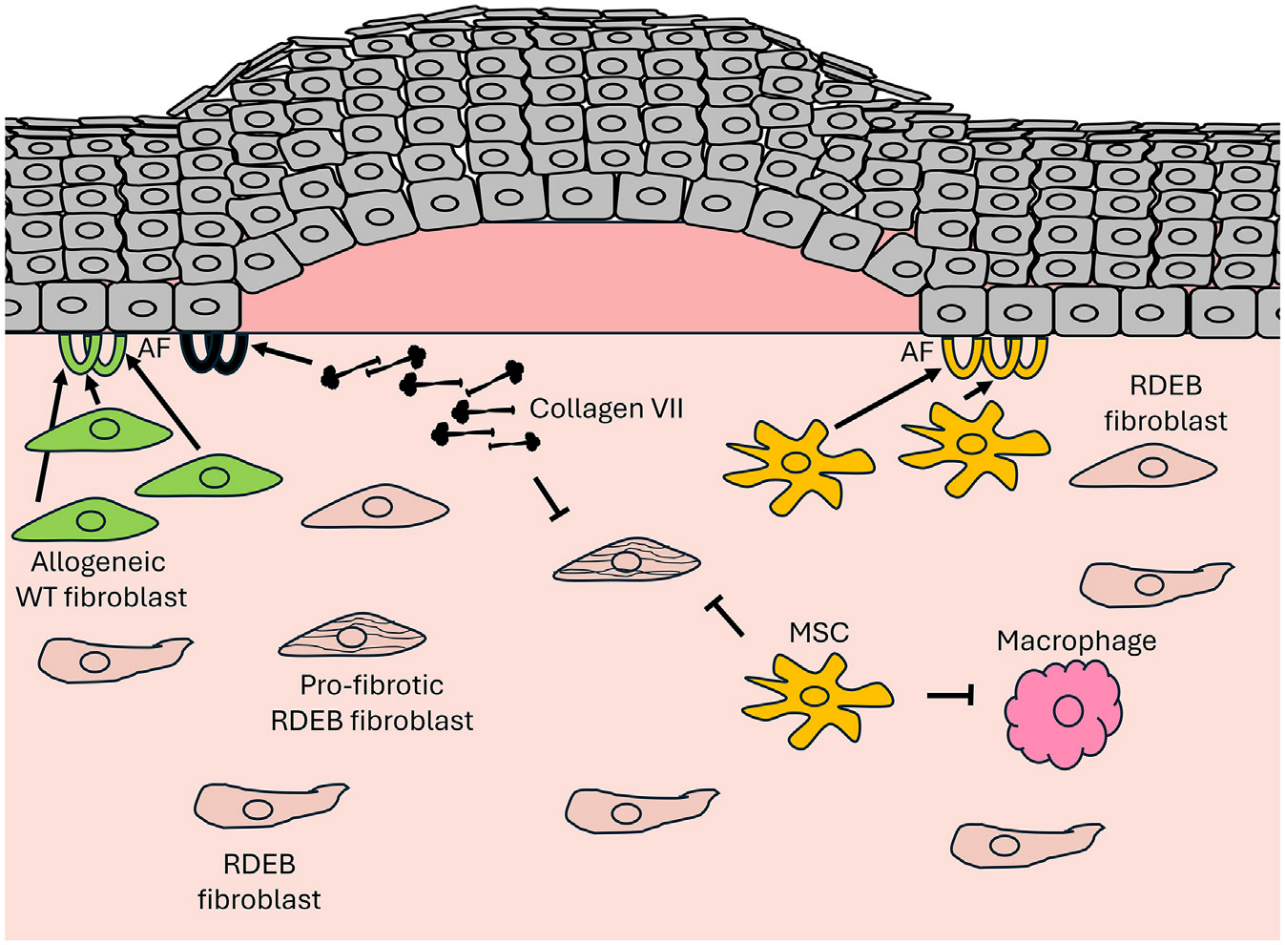
Schematic illustration of selected intracellular approaches Cell, gene, and protein therapies that restore dermal type VII collagen can improve skin cohesion while reducing inflammation and fibrosis in RDEB. The illustration depicts blistered RDEB skin, where fibroblasts become activated by injury and inflammation, promoting tissue contraction and fibrosis. Intradermally injected allogeneic fibroblasts (green) can serve as vehicles for type VII collagen replacement, restoring anchoring fibrils (AFs). Through paracrine effects, they may also support wound healing. Similarly, mesenchymal stromal cells (MSCs) (yellow), delivered at high concentrations intradermally, can provide type VII collagen to the epidermal basement membrane with subsequent AF formation occurring. MSCs also reduce inflammation and may limit fibroblast activity, a benefit that can be achieved by systemic activities and with significantly fewer cells when administered via intravenous infusion. Additionally, type VII collagen, whether delivered as a recombinant protein or through gene-expression restoration, not only promotes AFs and enhances skin cohesion but also mitigates pro-fibrotic activity in the skin.

A distinct advantage of MSCs in the treatment of genodermatoses affecting multiple organs, such as RDEB, is that they can be obtained from multiple anatomic sites and administered locally or systemically and migrate and differentiate into tissue-specific stromal cells within affected tissues. Interestingly, however, even intravenous injection of gene-corrected dermal RDEB fibroblasts has been shown to reach RDEB skin grafts in mice and promote type VII collagen deposition.^139^ This strategy has not been further pursued, and most subsequent studies on intravenously administered cells have focused on MSCs or specific MSC-like types or derivatives. In general, intravenous administration of cells, particularly MSCs, has demonstrated substantial and prolonged disease-modulating activity in RDEB (Figure 5). While it is clear that systemically administered MSCs may populate the skin and other tissues and contribute to type VII collagen replenishment,^140^^,^^141^ a contributing, and potentially primary, disease-modifying effect appears to be through the modulation of tissue inflammation and cellular activities, as indicated by preclinical^67^^,^^142^ and clinical trials.^138^^,^^141^^,^^143^^,^^144^ Such adaptive effects have also been proposed for fibroblasts that possess immune-modulating properties^145^ that may ameliorate fibrosis.

### Small molecules

#### Stop codon readthrough

A portion (∼15%–20%^146^) of *COL7A1* inactivating mutations are due to premature termination codons (PTC). Aminoglycosides, a class of antibiotics, possess the ability to mediate PTC readthrough by altering the conformation of ribosomal RNA^147^ and has been applied topically and intravenously for RDEB. Gentamicin ointment was given to five RDEB patients topically (0.1% compound, three times per day for 2 weeks) or 8 mg intradermally (daily for 2 days) and induced type VII collagen with deposition of AF.^148^ An open-label trial employing 7.5 mg/kg intravenously (daily for 2 weeks or twice weekly for 12 weeks) reported increased type VII collagen that persisted for at least 6 months and resulted in significant wound closure.^149^ While toxicity from these studies was not observed, gentamicin can exhibit neuro-,^150^ oto-,^151^ and nephrotoxicities.^152^ Toxicity, electrolyte imbalance, was observed following topical gentamicin in an 11-year-old with laminin-332-deficient junctional EB (JEB).^153^ As such, other readthrough-inducing drugs (RTID) have been pursued for epidermolysis bullosa. Ataluren is an RTID that competitively inhibits release factor complex termination activity^154^ and use in JEB was well tolerated and low levels of laminin β3 protein that is absent in JEB was observed.^153^ *In vitro* studies on *COL7A1* cDNA, however, showed limited readthrough efficacy of selected PTCs.^155^ Efforts continue for the identification of RTIDs with the use of large chemical library screens to identify compounds for use in EB.^156^ Since the readthrough efficacy can be influenced by the higher-order structure of pre-mRNA, screening would be most informative when performed under physiological expression conditions based on genomic DNA. Newly identified or repurposed RTIDs represent a pharmaceutical approach for restoring gene activity and while it may not be applicable to all genetic etiologies (insertions, deletions, or amino acid changes) it may synergize with other gene and cellular therapeutics to promote more complete responses. The concept of therapeutic agent synergy is important because the primary genetic driver of a given condition such as RDEB begets other cascading secondary (and tertiary) pathological manifestations that may not be relieved by gene restoration alone.

#### Secondary pathologies and combinatorial therapies

The dermal, mesenchymal cellular composition is naturally diverse, with multiple phenotypic and functional subpopulations that in disease states frequently contribute to disease progression and pathological manifestations of the genodermatoses. This also applies to cases in which only epidermally relevant genes are at fault.^9^ Such changes, evoked by epidermal or dermal damage responses, tend to lead to senescence-like changes in fibroblasts, resulting in a variety of complications, including atrophy and a microenvironment that accommodates tumor growth.^157^^,^^158^

#### Fibrosis

In diseases with deeper dermal instability, such as RDEB, the sustained fibroblast activation progresses to fibrosis. This contributes significantly to morbidity but also may increase challenges for effective delivery of vector- and cell-based therapeutics due to increasing physical constraints. It should also be considered that fibroblasts themselves, under such conditions, may become pathological and actively mediate or enable disease progression. In fibrosing conditions, fibroblasts can undergo accelerated proliferation, leading to increased fibroblast density and phenotypic shifts toward contractile myofibroblastic states.^159^ This poses a potential conundrum for treatments targeting the dermis and fibroblasts in genetic diseases associated with skin fibrosis. In such a state, is it beneficial to broadly restore the expression of the faulty gene in multiple cell types, or would the depletion of disease-promoting fibroblasts first, be necessary?

Studies to answer this question have shown that type VII collagen produced by a cell or provided as a recombinant protein can reduce certain fibrosis-related activities, such as contractility, angiogenesis, and the synthesis of ECM proteins that support fibrosis and tumor progression.^129^^,^^160^^,^^161^^,^^162^ This suggests that type VII collagen reintroduction might naturally reverse pathological fibroblast phenotypes—a hypothesis partially supported by preclinical work.^129^^,^^161^ Consequently, in this context of RDEB, the active depletion of pathological fibroblasts may not be necessary. Unrelated to fibrosis, a study has shown that processes required for anchoring fibril formation may be impaired in human RDEB skin and are not corrected by fibroblast administration alone. Supporting this, injured skin sites in RDEB mice display reduced abundance and activity of meprins—proteinases potentially involved in the maturation of type VII collagen necessary for the formation of stable AFs.^163^

#### Combination therapies

Such divergence as above raises an important consideration and becomes part of the treatment strategy calculus. Thus, additional considerations whereby gene correction alone may not resolve all manifestations and correcting the leading causes of morbidity and mortality must be accounted for with approaches toward better outcomes. Strategies with this concept in mind have been pursued using pharmaceuticals such as losartan. Losartan has long been used for hypertension and acts by blocking the angiotensin receptor and angiotensin II (Ang II) responses.^164^ Because it accomplishes this agnostic to the manner in which Ang II is generated, it has been applied for “off-label” purposes because of the pro-fibrotic and pathogenic role of Ang II in other conditions.^165^ We and others have pursued it for RDEB^166^^,^^167^^,^^168^^,^^169^^,^^170^^,^^171^ and in an open-label, single-arm, phase 1/2 trial (REFLECT-trial) the drug (EudraCT, 2015-003670-32), without compensatory type VII collagen rescue, was well tolerated and short-term results indicated disease stabilization and some improvement in pathology.^167^ It will be exciting to determine if losartan and provision of type VII collagen with one of the strategies we have detailed will synergize to address the full pathogenic spectrum of RDEB.

Apart from limiting fibrosis, a major advantage of using losartan in combination with a therapy with the intent to cure is that it reduces tissue inflammation.^168^ Reduction in inflammation might reduce the risk of adverse immune reactions toward the expressed transgene—in the case of RDB, type VII collagen—as it will lower the adjuvant activity of the tissue. Limiting tissue inflammatory activity in chronically unstable and damaged tissues through other means, e.g., by using glucocorticoids, delays progression of Duchenne muscular dystrophy.^172^^,^^173^ It is important to note that preconditioning with glucocorticoids can improve transgene expression and treatment efficacy.^174^ Thus, in the context of an injury- and inflammation-driven disease of the skin, such as RDEB, reducing inflammation and injury responses may not only delay fibrosis but also may improve the efficacy of type VII collagen restorative therapies. However, since drugs like glucocorticoids come with immune-depressing side effects and a functional immune system is essential in a disease with a high propensity for cancer and bacterial infections, careful studies are needed to determine the potential benefits of drug treatment combinations and optimal treatment regimens.

## Outlook

Although we have largely discussed studies on RDEB, this condition was chosen primarily as a paradigm to create a focused discussion and, importantly, because it is one of the genodermatoses in which significant advancements have been made using cell, gene, and/or pharmacological therapies. Importantly, this might not be the case for all diseases and extracellular factors in all contexts, even if both the major structural overlying and underlying cell types in basement membranes express them. One such example is the glomerular basement membrane and laminin-521.^175^ This is a basement membrane specialized to provide fluid filtration and firm anchoring, with podocytes and endothelial cells attaching to opposite sides of the structure. Laminin-521 is essential for the function of the glomerular basement membrane, and genetic deficiency in laminin-521 causes a nephropathy known as Pierson syndrome.^176^ Studies using systemic administration of recombinant laminin-521 in Pierson syndrome mice showed that laminin-521 does not cross from the endothelial to the podocyte side of the glomerular basement membrane.^175^ Thus, with regard to the glomerular basement membrane, the directionality of delivery is an important factor to consider for treatment approaches. Knowing the restriction of laminin-521 to pass through a basement membrane, it is unexpected that a protein of similar weight—type VII collagen, both approximately 900 kDa—can do this in the epidermal basement membrane. Underlying differences could include molecular and topographical differences in the organization of the epidermal and glomerular basement membranes.^176^ In other words, for gene and cell therapies of diseases affecting the basement membrane zone, it is important to consider tissue- and site-specific differences in the extracellular matrix restricting or facilitating movement of proteins. Thus, detailed molecular and biophysical knowledge of a tissue in a healthy and diseased state is needed for informed design of interventional gene or cellular therapies to include the potential use of augmentative small molecules.

Beyond the genodermatoses that are comparatively rare, the concepts we raise and delivery approaches/strategies have broader impact for higher prevalence conditions. Congenital melanocytic nevi are present in 1%–6% of neonates and most are caused by *NRAS* gene mutations.^177^ NRAS interfering RNAs have been developed,^178^ as have LNPs for skin siRNA delivery.^179^ Gene, cell, and LNP therapies have also been pursued for chronic wounds (e.g., diabetic ulcers) and burns that can occur frequently and among multiple age groups. Dermal scaffolds with polyethylenimine encapsulated plasmids encoding vascular endothelial growth factor and angiopoietin-1 can promote angiogenesis and wound healing.^180^ Further, LNPs have been applied as gene and drug delivery vehicles for enhanced burn wound healing^181^ and highlight the technological advancements from and with the genodermatoses for more expansive applicability.

## Conclusions

Monogenic diseases are attractive targets for developing gene and cell therapies and the accessibility of the skin makes it well suited for delivery technology development. We highlight approaches from the outside where genes, drugs, and cells can be applied topically, into, and through the dermis. We also accentuate strategies from the inside where therapeutics could be administered internally and rely on natural or engineered tropism for the desired compartment. With these, we also make note of the “middle,” which we define as the genotype and primary and secondary phenotypic disease manifestations. Disease-causing genotypes can be highly heterogeneous, making vector development and applicability across a number of patient cohorts a key consideration. The phenotype may extend beyond the disease-causing driver and lead to a cascading effect and an important factor in treatment is whether gene therapy alone will offer therapeutic benefit or will other agents be required for augmentation? Skin conditions can be fiscally burdensome to treat and can exhibit devastating manifestations that take a high toll on patients and their families. The continued development and advancement of cellular, pharmacological, and gene correction and delivery systems represent powerful additions to the therapeutic armamentarium.

Paraphrasing and borrowing from the philosopher Hegel’s dialectical method^182^ that describes a thesis, antithesis, and synthesis where problems (contradictions and conflicts) drive progress and lead to new solutions, we predict a challenge-driven solution continuum that will lead to not only improvements but cures. Doctor Tilbury Fox first described RDEB in 1879 and from then until the 21st century treatment was primarily palliative. As new technologies such as gene and cellular therapeutics emerged it offered efficacy but required specialized medical professionals and infrastructure. The properties of HSV-1 help alleviate the problem of resource and infrastructure-intensive treatments by enabling shipment closer to the patient and storage under refrigerated or room temperature conditions and topical, and repetitive, delivery by local medical personnel.^183^ This democratizes but does not economize the HSV-1/B-VEC treatment regimen and problems of cost, scale, and delivery over large surface areas will drive progress and promote new solutions. Initiatives and efforts are being catalyzed by organizations such as CaringCross whereby high budget impacts are balanced by the most important metric: high patient impacts.^184^

## Acknowledgments

A.N.’s research on the discussed topics is supported by grants NY90/6-1, CRC1160 project B03, and CRC1479 project identification: 441891347- P13 from the 10.13039/501100001659German Research Foundation (DFG). Work by T.M.R. and S.P. was partially supported by 10.13039/100004319Pfizer. M.J.O. and J.T. receive support from 10.13039/100000002NIH R01AR063070. M.J.O. receives further support from the 10.13039/100000865Bill and Melinda Gates Foundation, the 10.13039/100000885Children's Cancer Research Fund, the Kidz1stFund, and the Henry N. Jackson Foundation/US Department of Defense: PO 1071281 FMP# 6248 -HJF Award#: 67423.

Images in Figures 1, 2, 3, and 4 were created in BioRender (Toronto, Ontario).

## Author contributions

All authors contributed to the drafting and editing of the manuscript. A.N. and M.J.O. are equal contributors and are co-corresponding authors.

## Declaration of interests

The authors declare that they have no competing interests.
